# Bioactive Compounds Isolated from the Species of the *Campanula* Genus: A Review

**DOI:** 10.3390/molecules30234495

**Published:** 2025-11-21

**Authors:** Josiane Alhage, Abir Abdel Rahman, Charbel Al-Bayssari, Mohamad T. Raad

**Affiliations:** 1 Department of Biological and Chemical Sciences, School of Arts and Sciences, Lebanese International University, Mazraa, Beirut P.O. Box 146404, Lebanon; mohamad.raad01@liu.edu.lb; 2 Faculty of Natural and Applied Sciences, Notre Dame University, Zouk Mosbeh P.O. Box 72, Lebanon; 3 Department of Medical Laboratory, Faculty of Health Sciences, University of Balamand, Tripoli P.O. Box 100, Lebanon; abir.abdelrahman@balamand.edu.lb (A.A.R.); charbel.baysari@balamand.edu.lb (C.A.-B.)

**Keywords:** *Campanula*, phenolic acids, flavonoids, coumarins, acetylenic compounds, triterpenes, alkaloids

## Abstract

The *Campanula* genus (Campanulaceae) comprises nearly 300 herbaceous species widely distributed across the Northern Hemisphere. Beyond their ornamental value, many species have been traditionally employed to treat inflammatory, respiratory, and cardiovascular disorders. Phytochemical studies have revealed a remarkable diversity of bioactive constituents, including phenolic acids, flavonoids, coumarins, acetylenic compounds, triterpenes, and alkaloids. These metabolites exhibit broad pharmacological activities, such as antioxidant, anti-inflammatory, antimicrobial, cytotoxic, and cardioprotective effects. This review provides a comprehensive overview of the isolated compounds from the *Campanula* species, summarizing their chemical diversity, pharmacological mechanisms, and structure–activity relationships. It also highlights underexplored species and compound classes with potential therapeutic significance. By integrating the phytochemical evidence with pharmacological insights, this work underscores the value of the *Campanula* species as promising natural resources for future drug discovery and development.

## 1. Introduction

Medicinal plants have been universally used for centuries in various traditional medicine systems, as they contain a wide range of bioactive molecules, such as flavonoids, alkaloids, terpenoids, and essential oils, among others [1]. These bioactive molecules, extracted from different parts of the plant, often exhibit antioxidant, anti-inflammatory, antifungal, antibacterial, antiproliferative, and other therapeutic effects [2,3].

Phytochemical screening plays a vital role in the development of both traditional medicine applications and modern pharmaceutical research by validating the efficacy and safety of traditionally used treatments. Furthermore, such screening has led to the discovery of the bioactive molecules applied in agriculture, cosmetics, drugs, and food processing [4,5,6,7,8].

The genus *Campanula*, belonging to the Campanulaceae family, comprises approximately 300 herbaceous species, named for the bell-shaped blue flowers characteristic of most species. These species are widely distributed across Asia, the Black Sea, and Mediterranean regions [9]. Several species have a long history of traditional medicinal use: *Campanula glomerata*, *C. persicifolia*, *C. rotundifolia*, *C. bononiensis*, *C. sibirica*, and *C. patula* are employed in Russian folk medicine for treating epilepsy, nervous disorders, coughs, sore throats, headaches, rheumatism, and inflammation [10,11]. Similarly, *C. medium*, *C. cervicaria*, *C. rotundifolia*, *C. latifolia*, and *C. trachelium* are traditionally used in Italian medicine for comparable purposes [12].

From an ethnopharmacological perspective, these traditional uses correlate with the phytochemical composition of the species. The *Campanula* species are rich in phenolic acids, flavonoids, acetylenic compounds, and triterpenes, which are likely responsible for their reported anti-allergic, antioxidant, spasmolytic, antiviral, and antimicrobial activities [13]. Furthermore, geographic and taxonomic variations influence the distribution and abundance of these bioactive compounds, providing insight into their species-specific medicinal potential.

From a commercial standpoint, cultivation of the *Campanula* species is primarily for ornamental purposes [14]. However, understanding the link between phytochemistry, traditional uses, and geography can inform both conservation strategies and targeted pharmacological research.

The main objective of this study is to provide a comprehensive review of the phytochemistry and pharmacological applications of the *Campanula* genus. This review aims to support both the preservation of traditional knowledge and modern scientific discovery, serving as a foundation for future investigations into the medicinal potential of the *Campanula* species.

## 2. *Campanulaceae* Family

The Campanulaceae family includes 84 genera and 2400 species of dicotyledonous plants. The species of this family can be found on six continents and many oceanic islands. It is cosmopolitan in distribution, with representatives on six continents and many oceanic islands, and can grow in both cold and dry climatic conditions. The two main subfamilies are Campanuloideae, found mainly in temperate zones of the Old World, and Lobelioideae, distributed in the tropical and subtropical zones of the world.

The species of this family are usually herbaceous perennials, woody or herbaceous lianas that can twine and can be annuals or biennials. They can be described as subshrubs, shrubs, treelets, or pachycaul rosette plants, while the trees can reach up to 15 m tall. Most species are terrestrial, less often epiphytic or aquatic, and can have a milky latex that may be colored. Flowers of the species of this family are actinomorphic or zygomorphic, epigynous, and hermaphrodite. While the leaves are estipulate, alternate, infrequently whorled or opposite, often simple (pinnate when compound), entire, sessile, or petiolate, occasionally sheathed, and serrated in different ways The stems, acaulescent or rhizomatous, can be simple or highly branched woody or herbaceous lianas that can twine [15].

The major economic value of the Campanulaceae species is in horticulture. Many species are grown as ornamentals and are commonly available in the gardening trade. The best-known species in this area are perennial bellflowers (e.g., *Campanula persicifolia*, *Campanula carpatica*, *Campanula glomerata*). Other species, such as *Lobelia cardinalis*, *Platycodon grandiflorus*, and *Trachelium caeruleum*, are used for the same purposes. In addition to horticulture, some species are cultivated for their nutritional purposes, such as *Campanula rapunculus* and *Platycodon grandiflorus*, that are used as vegetables in Europe and Eastern Asia, respectively. On another hand, several pharmaceutical products are also supplied from species of this family. For example, Lobeline, isolated from *Lobelia inflata*, is a pyridine alkaloid used in the treatment of asthma, as a respiratory stimulant, and to combat smoking. Moreover, the roots of *Codonopsis*, mainly *C. pilosula*, are the source of dangshen used in traditional Chinese medicine. It is a popular, less expensive substitute for ginseng, used as a tonic for fatigue, loss of appetite, and weakness. Studies have shown that the crude extract of this species promotes digestion, strengthens the immune system, dilates peripheral blood vessels, and inhibits the activity of the adrenal cortex; however, the bioactive molecules responsible for these effects are yet to be isolated or identified [15]. The roots of *Platycodon grandiflorus* are an important Asian remedy, “jie-geng” or “kikyo”, used as an expectorant and cough suppressant. Studies have shown that it also has analgesic, antipyretic, anti-inflammatory, and antibacterial properties. The active ingredient is a saponin (platycodin or kikyosaponin) [16]. Many species of the Campanulaceae family have local uses in folk medicine, and particularly among non-industrialized populations, but they have not been studied in detail or exploited commercially due to the complexity of their constituents. For example, *Wahlenbergia marginata* is used in traditional Chinese medicine in the treatment of coughs, tuberculosis, heart diseases, and as a tonic [17].

Bioactive molecules, such as flavonoids [18], polyhydroxylated piperidine and pyrrolidine alkaloids [19], terpenes, sterols [20], and glycosylated polyacetylenes [21], have been isolated from various species of the Campanulaceae family.

## 3. *Campanula* Genus

The *Campanula* genus includes 300 herbaceous species. The species of this genus are distributed in the northern hemisphere of the world and especially in mountainous areas. They are present in meadows and subalpine regions; however, many species require full sun for optimal development. The name refers to the blue, bell-shaped flowers of most species. All these species are herbaceous, perennial, biannual, or annual. The corolla can be characterized as campanulate, infundibuliform (funnel-shaped), tubular, cupuliform (bowl-shaped), or rotate, mostly fused and varying in color from blue to violet or white (rarely yellow, red, or pink). The ovary is usually obconical or oblong–obconical with (2–)3–5 locules. Calyx teeth are usually longer than the ovary and they can alternate with appendages. Capsules are pendent or erect, dehiscing by pores or valves or, rarely, indehiscent [15,22,23].

Studies have shown that some *Campanula* species possess antioxidant, anti-allergic, antiphlogistic, spasmolytic, and antiviral properties [13,24,25].

Some species of the *Campanula* genus have been used in traditional medicine for the treatment of constipation, laryngitis, tonsillitis, warts, and bronchitis [26]. For example, *Campanula glomerata*, *C. persicifolia*, *C. rotundifolia*, *C. bononiensis*, *C. sibirica*, and *C. patula* have been used in Russian folk medicine to treat epilepsy, nervous diseases, coughs, sore throats. head, rheumatism, and inflammation [10,11]

*Campanula medium*, *C. cervicaria*, *C. rotundifolia*, *C. latifolia*, and *C. trachelium* are used in Italy for the same purposes [12].

In addition, the fresh roots of *Campanula sulphurea* Boiss. have been chewed for the treatment of lung and heart problems; their infusions have been used for ear inflammations [27].

From a commercial point of view, however, the *Campanula* species are mainly cultivated for ornamental purposes [14].

## 4. Bioactive Compounds Isolated from the Species of the *Campanula* Genus

Most bioactive molecules isolated from the *Campanula* genus, between 1970 and 1990, were found in the extracts obtained by extractions carried out by conventional methods using solvents of variable polarities and purifications were carried out by precipitation and/or by polyamide gel chromatography column or by preparative chromatography.

Commonly used solvents for extracting bioactive compounds from the *Campanula* species include trichloromethane, dichloromethane, ethanol, methanol, water, and sometimes a combination of these solvents. Techniques such as liquid–liquid extraction and Soxhlet extraction have been employed to extract the bioactive compounds from *Campanula* plants. Compounds have been identified using UV, IR, HPLC, and NMR spectroscopy [13,28,29,30,31].

Most of the compounds found in the *Campanula* genus belong to the family of phenolic compounds such as phenolic acids, flavonoids, and coumarins.

### 4.1. Phenolic Acids Isolated from the Species of the Campanula Genus

Fourteen compounds belonging to the phenolic acid family have been identified in the *Campanula* genus (Table 1). Among these, chlorogenic acid (**1**), isolated from six species of the *Campanula* genus (*C. cephalotes*, *C. glomerata*, *C. persicifolia*, *C. patula*, *C. rapunculoides* L., and *C. maleevii*), is recognized for its antioxidant, anti-inflammatory, antibacterial, antiviral, hypoglycemic, lipid-lowering, cardioprotective, antimutagenic, anticancer, and immunomodulatory activities [32].

3-*p*-Coumaroylquinic acid (**2**), known for its antibacterial and anti-inflammatory properties, has been isolated from four species (*C. cephalotes*, *C. glomerata*, *C. rapunculoides* L., and *C. persicifolia*).

Methyl caffeate (**3**), found in *C. cephalotes*, *C. glomerata*, and *C. rapunculoides* L., exhibits cytotoxic, antiproliferative, antimicrobial, anti-platelet, and antioxidant activities.

Caffeic acid (**4**) occurs in *C. rotundifolia*, *C. persicifolia*, and *C. rapunculoides* L., whereas *p*-coumaric acid (**5**) is found in *C. patula*, *C. rotundifolia*, *C. persicifolia*, and *C. rapunculoides* L. Both acids display antioxidant, antibacterial, cytotoxic, anti-inflammatory, and antiproliferative effects.

Ferulic acid (**6**) and syringic acid (**7**) have been isolated from *C. rotundifolia*, *C. persicifolia*, *C. persicifolia*, and *C. patula*, respectively. These compounds exhibit a broad spectrum of activities, including antioxidant, anti-inflammatory, cardioprotective, antibacterial, and anticancer effects.

Protocatechuic acid (**8**), known for its antioxidant, antibacterial, anticancer, antihyperlipidemic, antidiabetic, and anti-inflammatory properties, has been detected in *C. persicifolia*.

Vanillic acid (**9**), found in *C. persicifolia*, *C. patula*, and *C. rotundifolia*, possesses antioxidant, anti-inflammatory, immunostimulatory, neuroprotective, hepatoprotective, cardioprotective, and anti-apoptotic activities.

*p*-Hydroxybenzoic acid (**10**), isolated from *C. persicifolia*, *C. lactiflora*, and *C. medium*, exhibits antimicrobial, anti-atherogenic, and antioxidant properties.

Barbatosides A and B (**11**, **12**), detected in *C. barbata*, are classified as saponins and display analgesic and anti-inflammatory activities.

Barbatosides C and D (**13**, **14**), also isolated from *C. barbata*, have not been reported in other *Campanula* species. These compounds are unique to this species, although their pharmacological properties remain uninvestigated.

Nine of the isolated phenolic acids—chlorogenic acid (**1**), methyl caffeate (**3**), caffeic acid (**4**), *p*-coumaric acid (**5**), ferulic acid (**6**), syringic acid (**7**), protocatechuic acid (**8**), vanillic acid (**9**), and *p*-hydroxybenzoic acid (**10**)—demonstrate significant antioxidant properties, which likely contribute to the plants’ defense against oxidative stress. These compounds have been isolated from nine different *Campanula* species (*C. glomerata*, *C. patula*, *C. rapunculoides* L., *C. maleevii*, *C. persicifolia*, *C. cephalotes*, *C. rotundifolia*, *C. lactiflora*, and *C. medium*).

Table 1 provides a comprehensive summary of all phenolic acids isolated from the *Campanula* species along with their verified pharmacological activities.

#### 4.1.1. Flavonoids Isolated from the Species of the *Campanula* Genus

Flavonoids are the most common compounds in plants, and they are known for their wide range of pharmacological and therapeutical effects. Most compounds isolated from the *Campanula* genus belong to this family. To date, 60 flavonoids have been isolated from 27 species of the *Campanula* genus (*C. persicifolia*, *C. rotundifolia*, *C. lactiflora*, *C. medium*, *C. ossetica*, *C. isophylla*, *C. rotundifolia*, *C. patula*, *C. pyramidalis*, *C. maleevii*, *C. rapunculoides* L., *C. glomerata*, *C. bononiensis* L., *C. cephalates*, *C. kolenatiana*, *C. kemulariae*, *C. punctata*, *C. cephalotes*, *C. barbata*, *C. alliariifolia*, *C. biebersteiniana*, *C. choziatowskyi*, *C. oblongifolia*, *C. hypopolia*, *C. cephalates*, *C. carpatica*, and*a C. poscharskyana*).

Luteolin (**15**), along with eleven of its glucosides, have been found in the species of the *Campanula* genus. Luteolin (**15**) is a flavone known for its antioxidant, anti-inflammatory, antimicrobial, cancer chemotherapeutic and chemoprotective activities, cardioprotective, antidiabetic, neuroprotective, and anti-allergic activities.

Quercetin (**35**), a flavonol known for its anti-SARS-CoV-2, antioxidant, anticancer, antiaging, antiviral, and anti-inflammatory activities, has been isolated from one species of the *Campanula* genus (*C. medium*). Nine glucoside derivatives of quercetin have been detected in the *Campanula* genus as well.

Kaempferol (**45**) has been found in *C. medium*. Studies have shown that this common flavonol has anti-inflammatory, antioxidant, neuroprotective, cardioprotective, anti-obesity, anti-ulcer, antidiabetic, cosmetic use, anti-osteoporotic, and anticancer properties. Four derivatives of kaempferol have been detected from *Campanula* genus as well.

Rhamnetin (**50**) flavonol has been isolated from *C. cephalates* and *C. cephalotes*, and it possesses anti-inflammatory, antioxidant, cardioprotective, anti-atherosclerosis, and neuroprotective activities. Eight Rhamnetin glucosides have been found in the species of the *Campanula* genus as well.

Myricetin (**59**), along with four derivatives, were isolated from one species of the *Campanula* genus (*C. bononiensis* L.). Studies have shown that this flavonol exhibits a wide range of pharmacological activities, such as iron-chelating, antioxidant, anti-inflammatory, anticancer, immunomodulator, antimyocardial damage, antiviral, immune system regulation, oxidative stress reduction, amelioration of cardiac dysfunction, and arrhythmia.

Moreover, eighteen of the flavonoids isolated from the species of the *Campanula* species have not yet been detected in any other species, and are original for these following species of the *Campanula* genus:

Luteolin-7-*O*-glucosyl(6 → 1)-*O*-arabinoside (Rotundiside) (**18**) isolated from *C. rotundifolia.*
Patularoside (**20**), Acetylcynaroside (**24**), and Campanoside (**25**) isolated from *C. patula.*
Luteolin-7-*O*-glucoside-4′-*O*-glucoside (**27**), Luteolin-7-*O*-rutinoside-3′-*O*-glucoside (**28**), Luteolin-7-*O*-rutinoside-4′-*O*-glucoside (**30**), and Luteolin-7-*O*-neohesperidoside-4′-*O*-glucoside (**31**) isolated from *C. persicifolia.*
Quercetin-3-*O*-rhamnosyl-(1 → 4)-galactoside (**39**), Isorhamnetin-3-rhamnosyl(1 → 4) glucoside (**56**) isolated from *C. rapunculoides* L.
Quercetin-3-*O*-rhamnosyl-(1 → 4)-glucoside (**41**) isolated from *C. rapunculoides* L. and *C. bononiensis* L.
Quercetin-3,7-di-*O*-glucoside (**42**) isolated from *C. punctata* and *C. bononiensis* L.
Isorhamnetin-4′-*O*-(*p*-hydroxybenzoyl)-3,7-di-O-glucoside (**64**) isolated from *C. lactiflora*
Barbatoflavane (**68**) isolated from *C. barbata*.
Bisdeacylplatyconine (**69**) isolated from *C. isophylla* and *C. carpatica.*
Campanin (**72**) isolated from *C. medium*, *C. isophylla* and *C. carpatica.*
Rubrocampanin (**73**) and Purprocampanin (**74**) isolated from *C. medium.*

A list of flavonoid compounds and flavonoid glycosides isolated from this genus along with their known and proven pharmacological activities (when applicable) are presented in Table 2.

#### 4.1.2. Coumarins Isolated from the Species of the *Campanula* Genus

Three coumarins have been isolated from the species of the *Campanula* genus. Compounds belonging to this family have numerous therapeutic applications, including photochemotherapy, anti-tumor, and anti-HIV treatment. Some are central nervous system stimulants, others are antibacterial, anti-inflammatory, and anticoagulant agents [139]. Table 3 shows that, among the three isolated coumarins, esculetin (**75**) has been evaluated for its pharmacological activities, such as antioxidant, anti-tumor, anti-inflammatory, antibacterial, antidiabetic, immunomodulatory, and anti-atherosclerotic activities [140]. Fraxoside (**76**) has been detected in *C. alliariifolia* and *C. ochroleuca.* A study showed that this coumarin exhibits anticancer activity by providing high binding affinity toward the carbonic anhydrase IX (hCAIX) protein, which is a membrane-spanning metalloenzyme, encoded by the CA9 gene, and can lead to various carcinomas if upregulated [141].

Fraxetol (**77**) has been isolated from two species of the *Campanula* genus (*C. alliariifolia* and *C. ochroleuca*). This coumarin has been detected in other species such as *Fraxinus excelsior* L. [142], *Pentalinon andrieuxii* [143], *Stewartia pseudocamellia* [144], and *Xanthoceras sorbifolium* [145] as well; however, no study has been realized yet to detect its biological activities.

#### 4.1.3. Acetylenic Compounds Isolated from the Species of the *Campanula* Genus

Nine acetylenic compounds have been isolated from six species of the *Campanula* genus. Among these compounds, lobetyol (**80**), isolated from two species of this genus, is known for its antioxidant, antiviral, anti-inflammatory, and cell proliferation inhibitory capacities [103,146]. While lobetyolin (**81**), isolated from five species of this genus, is known for its anticancer, antioxidant, cardioprotective, and anti-inflammatory activities [147].

Among the isolated acetylenic compounds, six were original for the species of the *Campanula* genus:

- 9-(tetrahydropyran-2-yl)-nona-*trans*,*trans*-2,8-diene-4,6-diynol (**78**) and 9-(tetrahydropyran-2-yl)-trans-nona-8-ène-4,6-diynol (**79**) isolated from *C. glomerata*;
- tetradeca-6-ene-8,10-diyne-1,5,12-triol (**83**), tetradeca-6,13-diene-8,10-diyne-1,5,12-triol (**84**), and tetradeca-6,12-diene-8,10-diyne-1,5,14-triol (**85**) detected in *C. medium* and *C. pyramidalis*;
- tetradeca-4,12-diene-8,10-diyne-1,6,7-triol (**86**) found in *C. pyramidalis*.

However, despite their detection, research into their potential pharmacological properties is yet to be conducted.

Moreover, no study has been conducted yet to reveal the pharmacological effects of lobetyolinin (**82**), even though it has been isolated from *C. glomerata* and other species, such as *Lobelia chinensis* [148] and *Radix Codonopsis* [147].

A list of acetylenic compounds isolated from this genus along with their known and proven pharmacological activities (when applicable) is presented in Table 4.

#### 4.1.4. Triterpenes Isolated from the Species of the *Campanula* Genus

In addition, twelve compounds from the triterpene family have been isolated from eight species of the *Campanula* genus.

β-sitosterol (**87**), a common triterpene usually found in plants and known for a wide range of pharmacological activities, such as antimicrobial, anti-inflammatory, anticancer, antifertility, angiogenic, antioxidant, immunomodulatory, antidiabetic, and antinociceptive activities, has been isolated from four species of the *Campanula* genus (*C. cephalotes*, *C. punctata*, *C. dasyantha*, and *C. istriaca* Feer).

Daucosterol (**88**), found in *C. cephalotes*, *C. dasyantha*, *C. langsdorffiana*, and *C. lactiflora*, exhibits antioxidant, antidiabetic, hypolipidemic, anti-inflammatory, immunomodulatory, neuroprotective, and anticancer activities.

Stigmasterol (**89**) and ursolic acid (**90**) were isolated, respectively, from *C. cephalotes*, *C. istriaca* Feer, *C. persicifolia*, *C. rotundifolia*, and *C. patula.* Studies have revealed a wide range of biological activities of those two compounds, such as antibacterial, antioxidant, anti-inflammatory, anti-cardiovascular, and anticancer activities.

Oleanolic acid (**91**) has been detected in *C. langsdorffiana*, while β-amyrin acetate (**92**) and Urs-20(30)-en-3-β-ol (**93**) have been detected in *C. patula.* These acids have showed anti-inflammatory, antinociceptive, hepatoprotective, anti-allergic, and many other pharmacological properties (Table 5).

3-β-acetoxylup-20(30)-en-29-al (**94**), 3-β-acetoxylup-20(29)-ene (**95**), 3-acetylptiloepoxide (**96**), and Lactifloroside A (**97**), isolated from *C. lactiflora*, have not yet been isolated from any other species, but no research has been realized yet to detect their pharmacological properties. While Lactifloroside B (**98**), detected in *C. lactiflora*, was isolated from only one other species (*Phyteuma japonicum*), and it possesses anti-inflammatory activity [153].

A list of these triterpenes isolated from this genus along with their known and proven pharmacological activities is presented in Table 5.

**Table 5:** Triterpenes isolated from the species of the *Campanula* genus.

No.	Name	Structure	Species	Known Pharmacological Activities
**87**	β-sitosterol	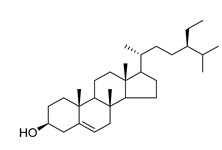	*C. punctata* [154]	Antimicrobial, anti-inflammatory, anticancer, antifertility, angiogenic, antioxidant, immunomodulatory, antidiabetic, and antinociceptive [155]
			*C. cephalotes* [154]	
			*C. dasyantha* [154]	
			*C. istriaca* Feer [156]	
**88**	Daucosterol	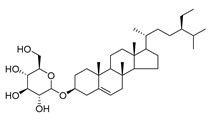	*C. punctata* [154]	Antioxidant, antidiabetic, hypolipidemic, anti-inflammatory, immunomodulatory, neuroprotective, and anticancer [157]
			*C. cephalotes* [154]	
			*C. dasyantha* [154]	
			*C. langsdorffiana* [154]	
			*C. lactiflora* [28]	
**89**	Stigmasterol	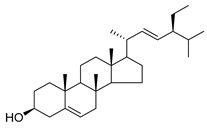	*C. cephalotes* [154]	Anticancer, anti-osteoarthritis, anti-inflammatory, antidiabetic, immunomodulatory, antiparasitic, antifungal, antibacterial, antioxidant, and neuroprotective [158]
			*C. istriaca* Feer [159]	
**90**	Ursolic acid	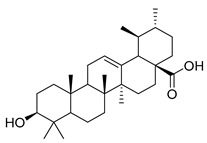	*C. persicifolia* [89]	Antimicrobial, anti-HIV, anti-HCV, antimalarial, anticancer agent; protective effect on lungs, kidneys, liver, and brain, anti-inflammatory, anabolic effects on skeletal muscles, and anti-osteoporosis [160]
			*C. rotundifolia* [75]	
			*C. patula* [159]	
			*C. istriaca* Feer [156]	
**91**	Oleanolic acid	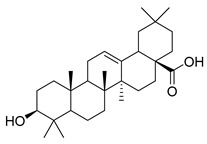	*C. langsdorffiana* [154]	Antidiabetic, antiviral, anti-HIV, antibacterial, antifungal, anticarcinogenic, anti-inflammatory, hepatoprotective, gastroprotective, hypolipidemic and anti-atherosclerotic activities, and anticancer [161]
**92**	β-amyrin acetate	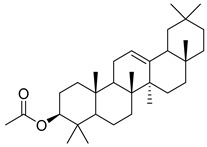	*C. patula* [159]	Anti-inflammatory [162], antinociceptive, hepatoprotective, and anti-allergic [163]
**93**	Urs-20(30)-en-3-β-ol	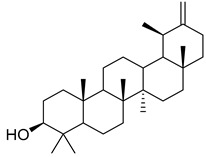	*C. patula* [164]	Anti-inflammatory, anti-oxidative, anti-carcinogenic, antiviral, and anti-allergic [165]
**94**	3-β-acetoxylup-20(30)-en-29-al	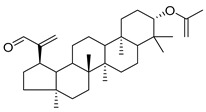	*C. lactiflora* [166]	-
**95**	3-β-acetoxylup-20(29)-ene	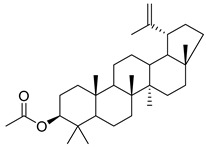	*C. lactiflora* [166]	-
**96**	3-acetylptiloepoxide	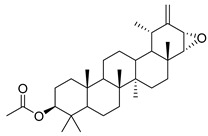	*C. lactiflora* [166]	-
**97**	Lactifloroside A	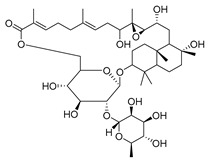	*C. lactiflora* [167]	-
**98**	Lactifloroside B	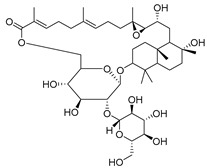	*C. lactiflora* [167]	Anti-inflammatory [153]

#### 4.1.5. Alkaloids Isolated from the Species of the *Campanula* Genus

Two alkaloids, (−)-Lobeline (**99**) and Campedin (**100**), have been isolated from the genus *Campanula* and specifically from the species *C. medium*. Among them, (−)-Lobeline (**99**) is used in the treatment of chronic bronchitis and asthmatic bronchitis, and it also inhibits dopamine uptake and promotes dopamine release from presynaptic storage vesicles [168]. Campedin (**100**) has only been detected in the *Campanula* genus, and no study has been conducted yet on its biological activities (Table 6).

**Table 6:** Alkaloids isolated from the species of the *Campanula* genus.

No.	Name	Structure	Species	Known Pharmacological Activities
**99**	(−)-Lobeline	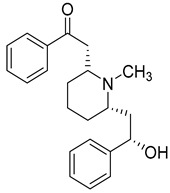	*C. medium* [169]	Treatment of chronic bronchitis and asthmatic bronchitis, inhibits dopamine uptake, and promotes dopamine release from presynaptic storage vesicles [168]
**100**	Campedin	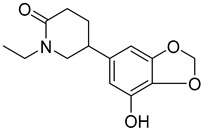	*C. medium* [169]	-

#### 4.1.6. Various Compounds Isolated from the Species of the *Campanula* Genus

Finally, five various compounds have been characterized from the species of the *Campanula* genus. Among them, inulin (**101**) has been isolated from *C. rapunculoides* L. Studies have shown that this compound has many biological activities, such as prebiotic effect, antioxidant, regulates sugar/lipid metabolism, promotes mineral absorption, reduces the risk of colon cancer, reduces the risk of obesity, regulates the immune system, antiviral, and anti-inflammatory.

While myo-inositol (**102**), known as a promotor of female fertility, mediator of cellular energetic metabolism, follicle maturation with menstrual cycle progression, and cellular motility, was detected in five species of the *Campanula* genus (*C persicifolia*, *C. elatior*, *C. taurica*, *C. hohenackeri*, and *C. cephalotes*).

Bis(2-ethylhexyl)adipate (**105**), isolated from *C. lactiflora*, has been identified as a toxic and irritant compound. However, this compound is original for the *Campanula* genus along with two other compounds, ethyl docosanoate (**104**) and 2-acetyl-myo-inositol (**103**), which have been respectively isolated from *C. lactiflora*, *C. rapunculoides*, *C. trachelium*, *C. pyramidalis*, *C. persicifolia*, *C. muralis*, *C. leutweinii*, *C. carpatica*, and *C. cephalotes.* Nevertheless, no study has been conducted yet to reveal the pharmacological effects of these two compounds (Table 7).

### 4.2. Mechanistic Overview of Reported Bioactivities

The *Campanula* genus is a rich source of structurally diverse bioactive compounds. Understanding their mechanisms of action is essential for further pharmacological development and potential therapeutic applications. Compounds isolated from the *Campanula* species exhibit a wide range of biological activities, including antioxidant, anti-inflammatory, antimicrobial, and anticancer effects. Elucidating their molecular mechanisms can provide valuable insights for integrating these compounds into modern medicine.

#### 4.2.1. Antioxidant Effects

Numerous compounds isolated from the *Campanula* species display potent antioxidant activity, including chlorogenic acid (**1**), methyl caffeate (**3**), caffeic acid (**4**), *p*-coumaric acid (**5**), ferulic acid (**6**), syringic acid (**7**), protocatechuic acid (**8**), vanillic acid (**9**), *p*-hydroxybenzoic acid (**10**), luteolin (**15**), luteolin-7-*O*-glucoside (**16**), luteolin-7-*O*-rutinoside (**17**), luteolin-7-*O-*primeveroside (cesioside) (**19**), graveobioside A (luteolin-7-*O*-glucopyranosido (2 → 1)-*O*-apio(D or L)furanoside (**21**), diosmin (**26**), chrysoeriol-7-*O*-rutinoside (**32**), vicinin-2 (**34**), quercetin (**35**), isoquercitrin (**36**), quercetin-3-*O*-galactoside (hyperoside) (**37**), quercetin-3-*O*-rutinoside (rutin) (**38**), kaempferol (**45**), kaempferol-3-*O*-glucoside (astragalin) (**46**), kaempferol-3-*O*-galactoside (trifolin) (**47**), rhamnetin (**50**), isorhamnetin-3-*O*-rutinoside (**55**), isorhamnetin-3-*O*-robinobioside (**57**), myricetin (**59**), myricetin-3-*O*-glucoside (**60**), myricetin-3-*o*-galactoside (**61**), cyanidin-3-*O*-glucoside (**65**), esculetin (**75**), lobetyol (**80**), lobetyolin (**81**), β-sitosterol (**87**), daucosterol (**88**), stigmasterol (**89**), and inulin (**101**).

The antioxidant activity of these compounds is mediated via multiple mechanisms:

1. Scavenging reactive oxygen species (ROS): Donating electrons or hydrogen atoms to neutralize hydroxyl radicals (•OH), superoxide anions (O_2_•−), and peroxyl radicals (ROO•), thereby preventing oxidative damage to lipids, proteins, and DNA [177]
2. Inhibition of lipid peroxidation: Preventing the formation of malondialdehyde and other toxic lipid peroxidation products, protecting cellular membranes [178].
3. Modulation of endogenous antioxidant enzymes: Upregulating superoxide dismutase (SOD), catalase (CAT), and glutathione peroxidase (GPx) to enhance intrinsic cellular defense against oxidative stress [179].
4. Metal ion chelation: Binding iron and copper ions to reduce metal-catalyzed ROS generation [180].
5. Regulation of gene expression: Upregulating antioxidant enzyme genes and downregulating pro-inflammatory genes, further mitigating oxidative damage [181].

#### 4.2.2. Anti-Inflammatory Effect

Compounds with demonstrated anti-inflammatory activity include chlorogenic acid (**1**), 3-*p*-coumaroylquinic acid (**2**), *p*-coumaric acid (**5**), ferulic acid (**6**), syringic acid (**7**), protocatechuic acid (**8**), vanillic acid (**9**), barbatoside A (**11**), barbatoside B (**12**), luteolin (**15**), luteolin-7-*O*-glucoside (**16**), luteolin-7-*O-*primeveroside (Cesioside) (**19**), diosmin (**26**), vicinin-2 (**34**), quercetin (**35**), kaempferol (**45**), kaempferol-3-*O*-glucoside (Astragalin) (**46**), kaempferol-3-*O*-rutinoside (**48**), rhamnetin (**50**), isorhamnetin-3-*O*-galactoside (**54**), myricetin (**59**), myricetin-3-*O*-galactoside (**61**), cyanidin-3-*O*-glucoside (**65**), esculetin (**75**), lobetyol (**80**), lobetyolin (**81**), β-sitosterol (**87**), daucosterol (**88**), stigmasterol (**89**), ursolic acid (**90**), oleanolic acid (**91**), β-amyrin acetate (**92**), urs-20(30)-en-3-β-ol (**93**), lactifloroside B (**98**), and inulin (**101**).

The anti-inflammatory mechanisms include the following:

1. Inhibition of pro-inflammatory mediators: Suppressing TNF-α, IL-1β, IL-6, IL-8, NO, and PGE2, which play central roles in the initiation and progression of inflammation [182].
2. NF-κB pathway modulation: Preventing NF-κB activation reduces transcription of pro-inflammatory genes, including iNOS, COX, and cytokines [183].
3. Inhibition of pro-inflammatory enzymes: Downregulation of cyclooxygenase (COX) and lipoxygenase (LOX) activities decreases mediator production [184].
4. MAPK pathway regulation: Attenuation of ERK, JNK, and p38 kinase activation diminishes inflammatory signaling [155].

#### 4.2.3. Antimicrobial and Antibacterial Effects

Bioactive compounds exhibiting antimicrobial activity include chlorogenic acid (**1**), 3-*p*-coumaroylquinic acid (**2**), methyl caffeate (**3**), caffeic acid (**4**), ferulic acid (**6**), syringic acid (**7**), protocatechuic acid (**8**), *p*-hydroxybenzoic acid (**10**), luteolin (**15**), patuloside (**22**), diosmin (**26**), quercetin-3-*O*-rutinoside (Rutin) (**38**), esculetin (**75**),β-sitosterol (**87**), stigmasterol (**89**), oleanolic acid (**91**), and ursolic acid (**90**).

Mechanisms of antimicrobial action include the following:

1. Disruption of microbial membranes and efflux systems.
2. Inhibition of DNA and RNA synthesis and function.
3. Interference with intermediary metabolism.
4. Induction of cytoplasmic coagulation.
5. Quorum sensing inhibition, which prevents microbial communication and biofilm formation [185].

#### 4.2.4. Antiproliferative and Cytotoxic Effects

Compounds showing antiproliferative activities include methyl caffeate (**3**), *p*-coumaric acid (**5**), luteolin-7-*O*-glucoside (**16**), kaempferol (**45**), isorhamnetin-3-*O*-rutinoside (**55**), and lobetyol (**80**).

Compounds with cytotoxic potential include chlorogenic acid (**1**), methyl caffeate (**3**), caffeic acid (**4**), syringic acid (**7**), protocatechuic acid (**8**), luteolin (**15**), luteolin-7-*O*-glucoside (**16**), patuloside (**22**), diosmin (**26**), chrysoeriol-7-*O*-rutinoside (**32**), apigenin-7-*O*-rutinoside (**33**), vicinin-2 (**34**), quercetin (**35**), isoquercitrin (**36**), quercetin-3-*O*-galactoside (hyperoside) (**37**), kaempferol (**45**), kaempferol-3-*O*-glucoside (astragalin) (**46**), myricetin (**59**), cyanidin-3-*O*-glucoside (**65**), delphinidin-3-glucoside (**67**), fraxoside (**76**), lobetyolin (**81**), β-sitosterol (**87**), daucosterol (**88**), stigmasterol (**89**), ursolic acid (**90**), oleanolic acid (**91**), and inulin (**101**).

Mechanisms of antiproliferative/cytotoxic effects include the following:

1. Induction of apoptosis: Activation of caspase-3 and -9, along with modulation of Bcl-2 family proteins, triggers programmed cell death [186].
2. Cell cycle arrest: Halting progression at G0/G1 or S phases allows DNA repair or promotes cancer cell death [187,188].
3. Inhibition of angiogenesis: Downregulation of VEGF and other pro-angiogenic factors reduces tumor vascularization and growth [189].
4. Anti-inflammatory synergy: Modulation of NF-κB and MAPK pathways contributes to suppression of cell proliferation and tumor progression [190].

## 5. Discussion

The *Campanula* genus is a rich source of diverse bioactive secondary metabolites, including flavonoids, phenolic acids, coumarins, triterpenes, and acetylenic compounds, while only two alkaloids have been reported to date. These compounds contribute to a wide range of biological activities, such as antioxidant, anti-inflammatory, antimicrobial, and antiproliferative/cytotoxic effects.

### 5.1. Critical Evaluation of Bioactivities

Antioxidant activity is one of the most consistently reported effects in the *Campanula* species, supported by multiple in vitro studies across various species and compounds (e.g., chlorogenic acid, quercetin, and kaempferol). Mechanisms include ROS scavenging, metal ion chelation, inhibition of lipid peroxidation, and modulation of endogenous antioxidant enzymes (SOD, CAT, GPx), providing a robust foundation for further in vivo validation.

Anti-inflammatory effects are also well-supported, particularly for compounds such as chlorogenic acid, luteolin derivatives, quercetin, kaempferol, and saponins (Barbatosides A–B). These compounds modulate NF-κB and MAPK signaling, inhibit pro-inflammatory cytokines (TNF-α, IL-1β, IL-6), and suppress enzymes such as COX and LOX, demonstrating reproducible pharmacological potential.

Antimicrobial and antibacterial activities have been demonstrated for several phenolic acids, flavonoids, and triterpenes (e.g., chlorogenic acid, caffeic acid, oleanolic acid, and ursolic acid). Mechanisms include membrane disruption, inhibition of DNA/RNA synthesis, interference with metabolism, and disruption of quorum sensing. These activities are reasonably well supported in vitro, but in vivo and clinical validation remain limited.

Antiproliferative and cytotoxic effects have been reported for multiple compounds (e.g., methyl caffeate, luteolin-7-*O*-glucoside, kaempferol, quercetin derivatives, myricetin, lobetyol, and ursolic and oleanolic acids). Mechanisms involve apoptosis induction, cell cycle arrest, and angiogenesis inhibition (VEGF downregulation). While these findings are promising, most evidence comes from in vitro assays, and further mechanistic and in vivo studies are required before drawing definitive conclusions.

### 5.2. Gaps and Limitations in the Literature

Despite significant advances, several gaps remain:

- Many *Campanula* species remain uninvestigated, leaving potential bioactive compounds undiscovered.
- Some isolated compounds, particularly Barbatosides C–D and rare triterpenes, lack pharmacological evaluation.
- Mechanistic studies are limited, especially for cardiovascular, cytotoxic, and neuroprotective effects.
- Dose–response relationships, long-term safety, and in vivo validations are often missing, limiting translation to clinical relevance.

### 5.3. Future Perspectives

Addressing these gaps requires the following:

1. Targeted phytochemical screening guided by observed bioactivities.
2. In vivo and mechanistic studies to validate preliminary in vitro results.
3. Exploration of understudied species for novel bioactive compounds.
4. Integration of modern pharmacological and molecular tools to elucidate mechanisms and therapeutic potential.

## 6. Materials and Methods

All ethnobotanical, phytochemical, and pharmacological information on the *Campanula* species was collected from online journals, books, and other scholarly sources published between 1951 and 2023. The following electronic databases were searched: Google, Google Scholar, ScienceDirect, PubMed, ResearchGate, and other relevant online collections.

The following keywords were used in various combinations:

- *Campanulaceae*, *Campanula*, *Campanula* species.
- Names of individual bioactive compounds described in this manuscript.
- Terms related to pharmacological activities of these compounds.

Inclusion criteria is as follows:

- Publications providing phytochemical, pharmacological, or ethnobotanical data on the *Campanula* species or its bioactive compounds.
- Articles published in English or with a reliable English translation.

Exclusion criteria is as follows:

- Studies not directly related to the *Campanula* species or its chemical constituents.
- Publications lacking sufficient experimental or observational data.

Data handling and synthesis are as follows:

- Duplicate records were manually removed.
- Relevant data were extracted and summarized for narrative synthesis.
- Quality assessment of included studies was not formally performed, as this is a narrative review.

PRISMA-style narrative for transparency is as follows:

- Total records identified through database searching: ~2450
- Records after duplicates removed: ~2180
- Records screened for relevance: ~2180
- Records excluded due to non-relevance: ~1640
- Full-text articles assessed for eligibility: ~540
- Studies included in the narrative synthesis: ~480

## 7. Conclusions

The present review demonstrates that the *Campanula* genus is a rich source of diverse bioactive secondary metabolites, including flavonoids, phenolic acids, coumarins, triterpenes, and acetylenic compounds, while only two alkaloids have been reported to date.

Evidence for antioxidant and anti-inflammatory activities is particularly robust, supported by multiple studies across different species and experimental models. By contrast, activities such as cytotoxic, antiproliferative, cardiovascular, and other therapeutic effects remain preliminary or speculative, often based solely on in vitro assays, and require further validation.

Despite progress, a significant number of the *Campanula* species remain understudied, and many isolated compounds lack detailed pharmacological evaluation or mechanistic understanding. Additional gaps include limited in vivo studies, dose–response data, and long-term safety assessments.

A new chemotaxonomic perspective highlights compound class enrichment in specific species and identifies taxa that may harbor novel bioactive metabolites. This insight can guide targeted phytochemical screening and prioritize species for future research.

In conclusion, a systematic exploration of the phytochemistry, pharmacology, and chemotaxonomy of the *Campanula* species could reveal novel bioactive molecules, strengthen mechanistic understanding, and support the development of new therapeutic agents, while addressing current knowledge gaps.

## Figures and Tables

**Table 1:** Phenolic acids isolated from the species of the *Campanula* genus.

No.	Name	Structure	Chemical Class	Species	Known Pharmacological Activities
**1**	Chlorogenic acid	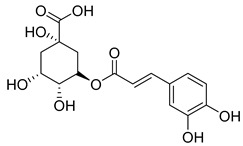	Phenolic acid	*C. cephalotes* [33]	Antioxidant, anti-inflammatory, antibacterial, antiviral, hypoglycemic, lipid lowering, anti-cardiovascular, antimutagenic, anticancer, immunomodulatory [32]
				*C. glomerata* [33]	
				*C. persicifolia* [29]	
				*C. patula* [34]	
				*C. rapunculoides* L. [35]	
				*C. maleevii* [36]	
**2**	3-*p*-coumaroylquinic acid	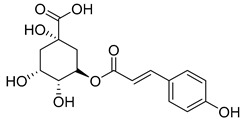	Phenolic acid	*C. cephalotes* [33]	Anti-bacterial [37], anti-inflammatory [38]
				*C. glomerata* [33]	
				*C. rapunculoides* L. [35]	
				*C. persicifolia* [29]	
**3**	Methyl caffeate	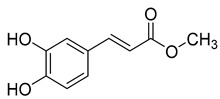	Phenolic acid	*C. cephalotes* [33]	Cytotoxic, antiproliferative [39], antimicrobial [40], anti-platelet activity [41], oxidative stress inhibiting activity [42]
				*C. glomerata* [33]	
				*C. rapunculoides* L. [35]	
**4**	Caffeic acid	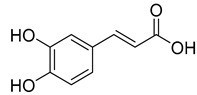	Phenolic acid	*C. rotundifolia* [29]	Antioxidant [43], antibacterial [44], cytotoxic [45,46]
				*C. persicifolia* [29]	
				*C. rapunculoides* L. [35]	
**5**	*p*-coumaric acid	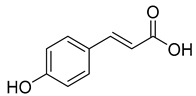	Phenolic acid	*C. rotundifolia* [29]	Antioxidant [47], anti-inflammatory [48], antiproliferative [49,50]
				*C. persicifolia* [29]	
				*C. rapunculoides* L. [35]	
				*C. patula* [34]	
**6**	Ferulic acid	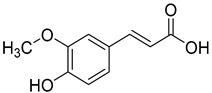	Phenolic acid	*C. rotundifolia* [29]	Antioxidant [51], anti-fibrosis effect [52], anti-inflammatory [53], anti-cardiovascular [54], antiplatelet [55], antibacterial [56], antiapoptotic [57]
				*C. persicifolia* [29]	
**7**	Syringic acid	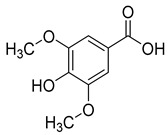	Phenolic acid	*C. persicifolia* [29]	Antidiabetic, anti-cardiovascular, anticancer, anticerebral ischemia; antioxidant, antimicrobial, anti-inflammatory, antiendotoxic, neuro and hepatoprotective activities [58]
				*C. patula* [34]	
**8**	Protocatechuic acid	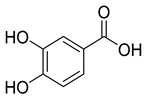	Phenolic acid	*C. persicifolia* [29]	Antioxidant, antibacterial, anticancer, antihyperlipidemic, antidiabetic, and anti-inflammatory [59]
**9**	Vanillic acid	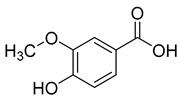	Phenolic acid	*C. persicifolia* [29]	Antioxidant, anti-inflammatory, immuno-stimulating, neuroprotective, hepatoprotective, cardioprotective, and antiapoptotic [60]
				*C. patula* [34]	
				*C. rapunculoides* L. [35]	
**10**	*p*-hydroxybenzoic acid	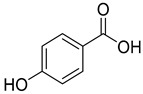	Phenolic acid	*C. persicifolia* [29]	Antimicrobial, anti-atherogenic, antioxidant [61]
				*C. lactiflora* [28]	
				*C. medium* [62]	
**11**	Barbatoside A (wahlenbergioside-3′-*O*-glucoside)	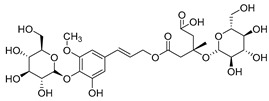	Saponin	*C. barbata* [31]	Analgesic, anti-inflammatory [63]
**12**	Barbatoside B (wahlenbergioside-3′-*O*-(2‴-(*p-*methoxycinnamoyl)- glucoside)	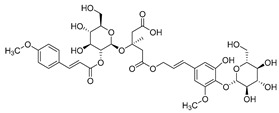	Saponin	*C. barbata* [31]	Analgesic, anti-inflammatory [63]
**13**	Barbatoside C (wahlenbergioside-3′-*O*-(4‴-(*transp*- coumaroyl)-glucoside)	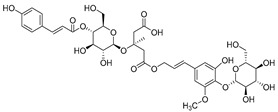	Phenylpropanoid	*C. barbata* [31]	Not yet investigated
**14**	Barbatoside D (wahlenbergioside-3′-*O*-(4‴-(*cis*-*p-*coumaroyl)- glucoside)	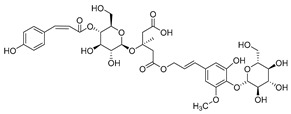	Phenylpropanoid	*C. barbata* [31]	Not yet investigated

**Table 2:** Flavonoids isolated from the species of the *Campanula* genus.

No.	Name	Structure	Species	Known Pharmacological Activities
**15**	Luteolin	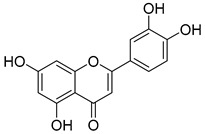	*C. persicifolia* [64]	Antioxidant, anti-inflammatory, antimicrobial, cancer chemotherapeutic and chemoprotective activities, cardioprotective, antidiabetic, neuroprotective, and anti-allergic [65]
			*C. rotundifolia* [64]	
			*C. lactiflora* [66]	
			*C. medium* [67]	
**16**	Luteolin-7-*O*-glucoside	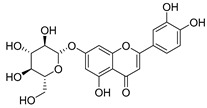	*C. lactiflora* [68]	anti-remodeling, antihypertensive, anti-inflammatory [69], antioxidation, antiproliferation, anticancer, and chemopreventive [70]
			*C. ossetica* [18]	
			*C. isophylla* [71]	
			*C. persicifolia* [72]	
			*C. rotundifolia* [64]	
**17**	Luteolin-7-*O-*rutinoside	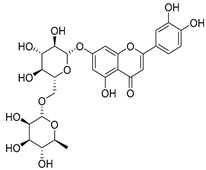	*C. persicifolia* [72]	Antioxidant [73] and antimutagenic effects [74]
			*C. rotundifolia* [75]	
**18**	Luteolin-7-*O*-glucosyl(6 → 1)-*O*-arabinoside (Rotundiside)	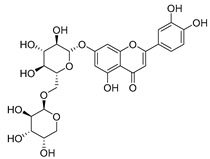	*C. rotundifolia* [76]	-
**19**	Luteolin-7-*O-* primeveroside (Cesioside)	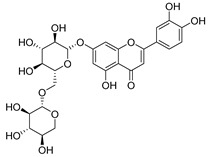	*C. rotundifolia* [76]	Antioxidant and anti-inflammatory [77]
**20**	Patularoside	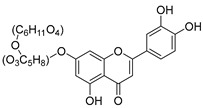	*C. patula* [34]	-
**21**	Graveobioside A (Luteolin-7-*O*-glucopyranosido (2 → 1)-*O*-apio(D or L)furanoside	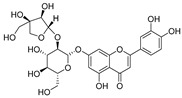	*C. patula* [78]	Antioxidant [79]
**22**	Patuloside	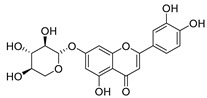	*C. patula* [80]	Anti-aging effects [81], antibacterial, and cytotoxic [82]
**23**	Luteolin-7-O-gentiobioside	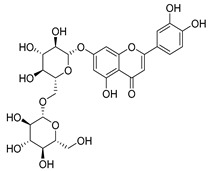	*C. rotundifolia* [75]	-
**24**	Acetylcynaroside	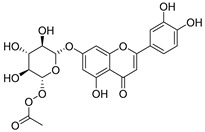	*C. patula* [83]	-
**25**	Campanoside	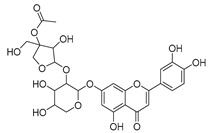	*C. patula* [84]	-
**26**	Diosmin	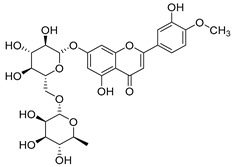	*C. patula* [85]	Anticancer, antidiabetic, antibacterial, anti-cardiovascular, liver protection, neuroprotection, antioxidative, antihyperglycemic, anti-inflammatory, antimutagenic, and anti-ulcer properties [86]
			*C. persicifolia* [87]	
			*C. pyramidalis* [88]	
**27**	Luteolin-7-*O*-glucoside-4′-*O*-glucoside	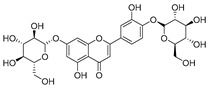	*C. persicifolia* [72]	-
**28**	Luteolin-7-*O*-rutinoside-3′-*O*-glucoside	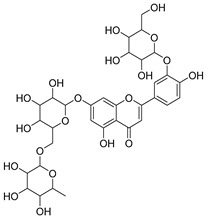	*C. persicifolia* [89]	-
**29**	Luteolin-4′-*O*-glucoside	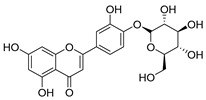	*C. persicifolia* [87]	Hyperuricemia and gout therapeutic effects [90]
**30**	Luteolin-7-*O*-rutinoside-4′-*O*-glucoside	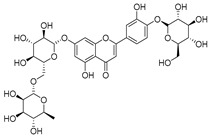	*C. persicifolia* [91]	-
**31**	Luteolin-7-*O*-neohesperidoside-4′-*O*-glucoside	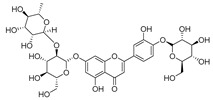	*C. persicifolia* [91]	-
**32**	Chrysoeriol-7-*O*-rutinoside	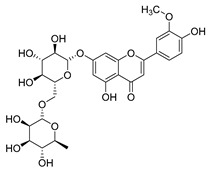	*C. persicifolia* [89]	Antioxidant [92] and cytotoxic effect [93]
**33**	Apigenin-7-*O*-rutinoside	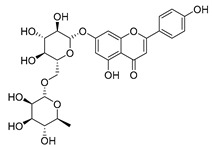	*C. persicifolia* [89]	Antimutagenic [94] and anticancer [95]
**34**	Vicinin-2	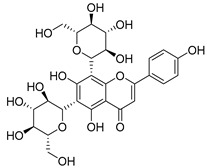	*C. persicifolia* [87]	Antioxidant, anti-inflammatory, anticancer, hepatoprotective, and antidiabetic [96]
**35**	Quercetin	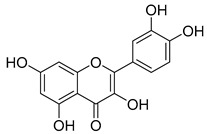	*C. medium* [67]	Anti-SARS-CoV-2, antioxidant, anticancer, antiaging, antiviral, and anti-inflammatory activities [97]
**36**	Isoquercitrin	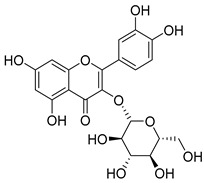	*C. persicifolia* [98]	Antioxidative, anticancer, cardioprotective, antidiabetic, and anti-allergic [99]
			*C. maleevii* [36]	
			*C. rapunculoides* L. [35]	
			*C. glomerata* [33]	
			*C. bononiensis* L. [100]	
			*C. cephalates* [33]	
			*C. kolenatiana* [18]	
			*C. kemulariae* [18]	
			*C. punctata* [101]	
			*C. cephalotes* [102]	
			*C. barbata* [31]	
			*C. alliariifolia* [103]	
**37**	Quercetin-3-*O*-galactoside (Hyperoside)	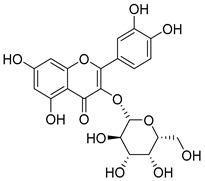	*C. kolenatiana* [18]	Antioxidant, analgesic, cytotoxic, nephrotoxic [104], and anti-HBV [105]
			*C. kemulariae* [18]	
			*C. biebersteiniana* [106]	
			*C. rapunculoides* L. [35]	
			*C. bononiensis* L. [100]	
			*C. glomerata* [33]	
**38**	Quercetin-3-*O*-rutinoside (Rutin)	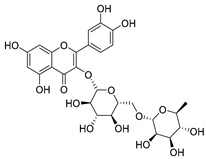	*C. persicifolia* [98]	Antioxidant, cytoprotective, vasoprotective, anticarcinogenic, neuroprotective, cardioprotective, antimicrobial, antifungal, anti-allergic, larvicidal, antiviral, immune boost effects, and organ protective effects [107]
			*C. maleevii* [36]	
			*C. choziatowskyi* [18]	
			*C. glomerata* [108]	
			*C. barbata* [31]	
			*C. rapunculoides* L. [35]	
			*C. bononiensis* L. [100]	
			*C. oblongifolia* [109]	
			*C. alliariifolia* [103]	
**39**	Quercetin-3-*O*-rhamnosyl-(1 → 4)-galactoside	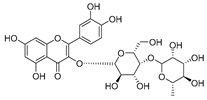	*C. rapunculoides* L. [35]	-
**40**	Bio-Quercetin	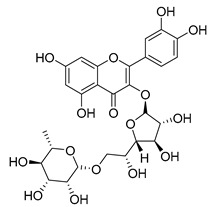	*C. rapunculoides* L. [35]	-
			*C. bononiensis* L. [100]	
			*C. glomerata* [108]	
**41**	Quercetin-3-*O*-rhamnosyl-(1 → 4)-glucoside	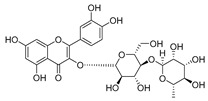	*C. rapunculoides* L. [35]	-
			*C. bononiensis* L. [100]	
**42**	Quercetin-3,7-di-*O*-glucoside	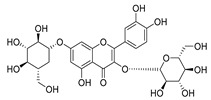	*C. punctata* [101]	-
			*C. bononiensis* L. [100]	
**43**	Quercetin-3-*O*-rutinoside-7-*O*-glucoside (Morkotin A)	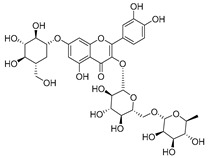	*C. persicifolia* [98]	-
**44**	Quercetin-3-*O*-galactoside-7-*O*-glucoside	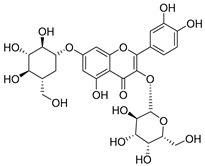	*C. rapunculoides* L. [35]	-
**45**	Kaempferol	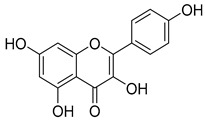	*C. medium* [67]	Anti-inflammatory, antioxidant, neuroprotective [110], anticancer, anti-osteoarthritis, antidepressive, antiproliferation, antidiabetic, and cytoprotective [111]
**46**	Kaempferol-3-*O*-glucoside (Astragalin)	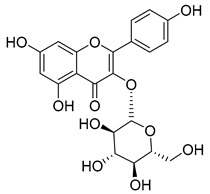	*C. punctata* [101]	Anti-inflammatory, antioxidant, neuroprotective, cardioprotective, anti-obesity, anti-ulcer, antidiabetic, cosmetic use, anti-osteoporotic, and anticancer [112]
			*C. barbata* [31]	
			*C. hypopolia* [113]	
			*C. alliariifolia* [103]	
**47**	Kaempferol-3-*O*-galactoside (Trifolin)	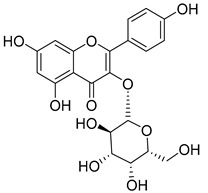	*C. Rapunculoides* L. [35]	Antioxidant activity [114]
			*C. glomerata* [33]	
			*C. bononiensis* L. [100]	
			*C. hypopolia* [113]	
**48**	Kaempferol-3-*O*-rutinoside (Nicotiflorin)	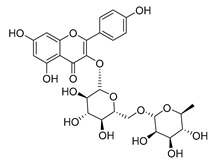	*C. bononiensis* L. [100]	Decreases arterial blood pressure and heart rate, hepatoprotective effects, antioxidant, anti-inflammatory, antinociceptive, antihypertensive and anti-anaphylactic effects, protective effect against cerebral ischemic damage and against memory dysfunction and oxidative stress [115]
			*C. barbata* [31]	
**49**	Kaempferol-3-*O*-robinobioside	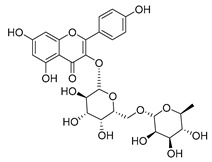	*C. bononiensis* L. [100]	Antiviral [116]
**50**	Rhamnetin	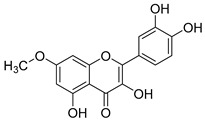	*C. cephalates* [33]	Anti-inflammatory [117], antioxidant, cardioprotective, anti-atherosclerosis, and neuroprotective [118,119]
			*C. cephalotes* [120]	
**51**	Rhamnetin-3-*O*-glucoside	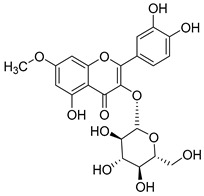	*C. cephalates* [33]	-
			*C. cephalotes* [102]	
**52**	Rhamnetin-3-*O*-galactoside	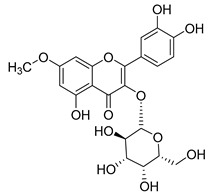	*C. rotundifolia* [29]	-
			*C. cephalates* [33]	
			*C. cephalotes* [120]	
**53**	Isorhamnetin-3-*O*-glucoside	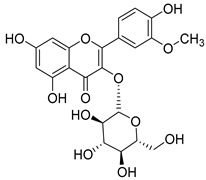	*C. rapunculoides* L. [35]	α-Glucosidase inhibitory activity [121]
			*C. glomerata* [33]	
			*C. bononiensis* L. [100]	
**54**	Isorhamnetin-3-*O*-galactoside	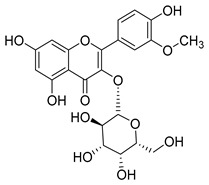	*C. glomerata* [108]	Antithrombotic [122] and anti-inflammatory [123]
**55**	Isorhamnetin-3-*O*-rutinoside	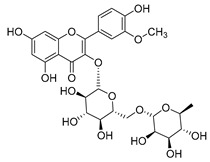	*C. glomerata* [108]	Antioxidant and antiproliferative [124]
**56**	Isorhamnetin-3-rhamnosyl(1 → 4) glucoside (Calendoside I)	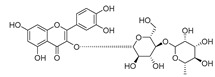	*C. rapunculoides* L. [35]	-
**57**	Isorhamnetin-3-*O*-robinobioside	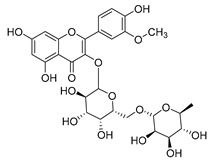	*C. glomerata* [109]	Antioxidant and antigenotoxic [125]
**58**	Isorhamnetin-3-*O*-glucuronoside	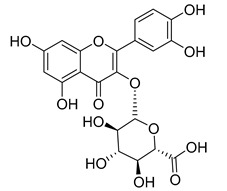	*C. glomerata* [126]	-
**59**	Myricetin	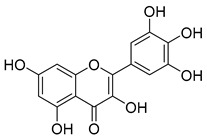	*C. bononiensis* L. [127]	iron-chelating, antioxidant, anti-inflammatory, anticancer [128], immunomodulator, antimyocardial damage, anti-viral, immune system regulation, oxidative stress reduction, amelioration of cardiac dysfunction, and arrhythmia [129]
**60**	Myricetin-3-*O*-glucoside	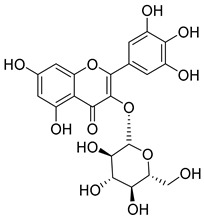	*C. bononiensis* L. [100]	Antioxidant and antidiabetic [130]
**61**	Myricetin-3-*O*-galactoside	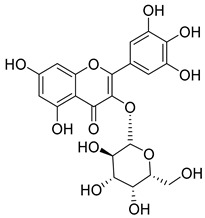	*C. bononiensis* L. [100]	Antinociceptive, anti-inflammatory [131], antigenotoxic, and antioxidant [132]
**62**	Myricetin-3-*O*-rutinoside	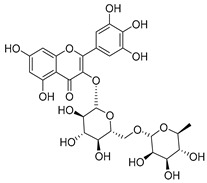	*C. bononiensis* L. [100]	-
**63**	Myricetin-3-*O*-robinobioside	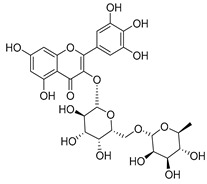	*C. bononiensis* L. [100]	-
**64**	Isorhamnetin-4′-*O*-(*p*-hydroxybenzoyl)-3,7-di-O-glucoside	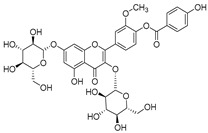	*C. lactiflora* [28]	-
**65**	Cyanidin-3-*O*-glucoside	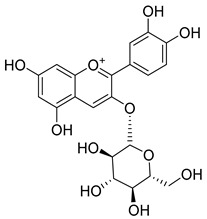	*C. glomerata* [21]	Antioxidant, anti-inflammatory, anticancer [133] DNA-Radical scavenging, gastroprotective, anti-inflammatory, anti-thrombotic chemopreventive, epigenetic factor, exerts protection against *Helicobacter pylori* infection, anti-age-related diseases, antidiabetic, and anti-cardiovascular [134]
**66**	Cyanidin-3-*O*-rutinoside	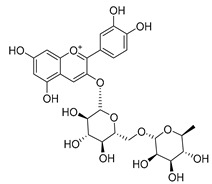	*C. glomerata* [21]	Antidiabetic [135]
**67**	Delphinidin-3-glucoside	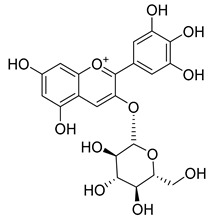	*C. isophylla* [71]	Inhibits platelet activation [136] and anticancer [137]
**68**	Barbatoflavane	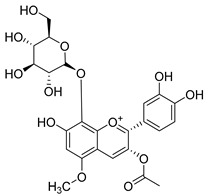	*C. barbata* [31]	-
**69**	Bisdeacylplatyconine	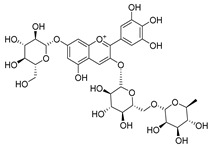	*C. isophylla* [71]	-
			*C. carpatica* [138]	
**70**	Deacylcampanin	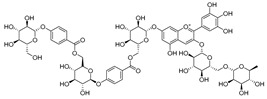	*C. isophylla* [138]	-
			*C. carpatica* [138]	
**71**	Violdelphin	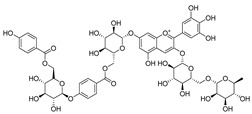	*C. poscharskyana* [138]	-
			*C. isophylla* [71]	
**72**	Campanin	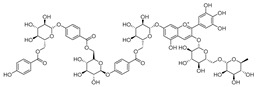	*C. medium* [62]	-
			*C. isophylla* [71]	
			*C. carpatica* [138]	
**73**	Rubrocampanin	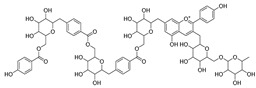	*C. medium* L. [62]	-
**74**	Purprocampanin	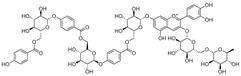	*C. medium* L. [62]	-

**Table 3:** Coumarins isolated from the species of the *Campanula* genus.

No.	Name	Structure	Species	Known Pharmacological Activities
**75**	Esculetin	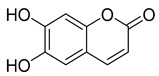	*C. rotundifolia* [29]	Antioxidant, anti-tumor, anti-inflammatory, antibacterial, antidiabetic, immunomodulatory, and anti-atherosclerotic [140]
			*C. persicifolia* [29]	
**76**	Fraxoside	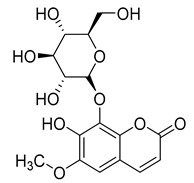	*C. alliariifolia* [30]	Anticancer activity [141]
			*C. ochroleuca* [30]	
**77**	Fraxetol	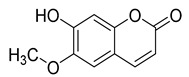	*C. alliariifolia* [30]	-
			*C. ochroleuca* [30]	

**Table 4:** Acetylenic compounds isolated from the species of the *Campanula* genus.

No.	Name	Structure	Species	Known Pharmacological Activities
**78**	9-(tetrahydropyran-2-yl)-nona-*trans*,*trans*-2,8-diene-4,6- diynol	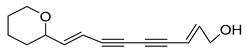	*C. glomerata* [149]	-
**79**	9-(tetrahydropyran-2-yl)-trans-nona-8-ène-4,6-diynol	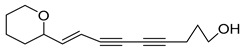	*C. glomerata* [149]	-
**80**	Lobetyol	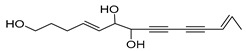	*C. glomerata* [21]	Antioxidant, antiviral, anti-inflammatory, and cell proliferation inhibitory capacities [103,146]
			*C. alliariifolia* [103]	
**81**	Lobetyolin	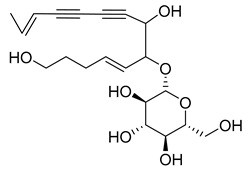	*C. glomerata* [21]	Anticancer, antioxidant, cardioprotective, and anti-inflammatory [147]
			*C. lactiflora* [150]	
			*C. medium* [151]	
			*C. alliariifolia* [103]	
**82**	Lobetyolinin	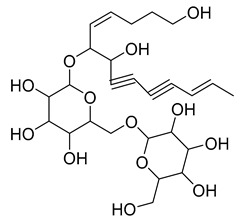	*C. glomerata* [21]	-
**83**	Tetradeca-6-ene-8,10-diyne-1,5,12-triol	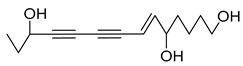	*C. pyramidalis* [152]	-
			*C. medium* [152]	
**84**	Tetradeca-6,13-diene-8,10-diyne-1,5,12-triol	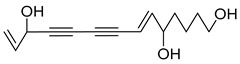	*C. pyramidalis* [152]	-
			*C. medium* [152]	
**85**	Tetradeca-6,12-diene-8,10-diyne-1,5,14-triol	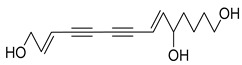	*C. pyramidalis* [152]	-
			*C. medium* [152]	
**86**	Tetradeca-4,12-diene-8,10-diyne-1,6,7-triol	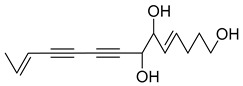	*C. pyramidalis* [152]	-

**Table 7:** Various compounds isolated from the species of the *Campanula* genus.

No.	Name	Structure	Species	Known Pharmacological Activities
**101**	Inulin	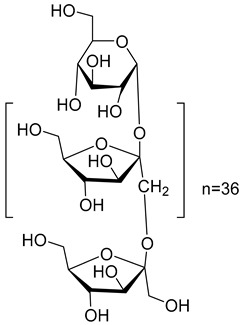	*C. rapunculoides* L. [170]	Prebiotic effect, antioxidant, regulates sugar/lipid metabolism, promotes mineral absorption, reduces the risk of colon cancer, reduces the risk of obesity, regulates the immune system, antiviral, and anti-inflammatory [171]
**102**	Myo-inositol	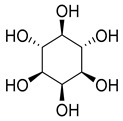	*C. persicifolia* [89]	Promotion of female fertility, mediator of cellular energetic metabolism, follicle maturation with menstrual cycle progression, and cellular motility [172]
			*C. elatior* [173]	
			*C. taurica* [173]	
			*C. hohenackeri* [173]	
			*C. cephalotes* [174]	
**103**	2-acetyl-myo-inositol	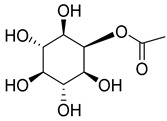	*C. rapunculoides* [175]	-
			*C. trachelium* [175]	
			*C. pyramidalis* [175]	
			*C. persicifolia* [175]	
			*C. muralis* [175]	
			*C. leutweinii* [175]	
			*C. carpatica* [175]	
			*C. cephalotes* [174]	
**104**	Ethyl docosanoate	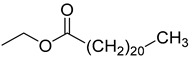	*C. lactiflora* [28]	-
**105**	Bis(2-ethylhexyl)adipate	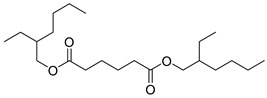	*C. lactiflora* [28]	Toxic and irritant [176]

## Data Availability

No new data were created or analyzed in this study. Data sharing is not applicable to this article.
